# In Toe-Toe

**Published:** 1869-11

**Authors:** 


### CORRESPONDENCE.

### ARTICLE V.

### *In Toe-Toe.*

" The American Journal's toe is worse—that is, it (the American Journal's toe) " whines louder," (Louder than what, thunder ? ) And we have still stronger evidence that its " the toe's " Editor is our old schoolmate Charlie * * under an assumed name." * * * When a new scholar made his appearance, Charlie alway maintained that *he* came to school only to step on *his* toe, especially if *he* had shoes on." * * * When we laughed at him he thought each one (of us) did it ' to conceal *his* vexation ' because *we* had to toe the mark and he *hadn't !* "

The above is a specimen taken from the first eight lines of an article written by that facetious, sarcastic, funny, dogmatical, rhetorical, *grammatical* editor of the the *Register*, and is a fair sample of the remaining portion of the article.

This Editor is a perfect Achilles; and like that irascible hero has *one* vulnerable point. But this is even more sensitive than Achilles' *heel* or Charlie's *toe.* "Bad grammar" is the most effective weapon, if you would vanquish this almost invulnerable editor. As Ithurial's spear was to Satan, so is "bad grammar" to this captious editor of the *Register*, only more so. Hurl this formidable weapon at him and he is overpowered to such a degree that you might "brain him with his lady's fan."

So keen are his grammatical perceptions, that the slightest deviation from the strict rules of Murray is instantly detected, and the article containing it condemned and *tortured.* For example:—A few months since one of the professors of Rush Medical College, delivered an address at a meeting of the officers or corporators of the Chicago Dental College, which his friends thought would interest the profession at large. They procured a copy which was sent to the editors of the *Register* for publication. But, no matter what the merits of the address were, or how much of value to the profession it might contain; the critical editor applies his grammatical touch-stone, when lo! By this infalible test he discovers "bad grammar." The address is refused and abused. No bad grammar must be found in the columns of the *Register!*—at least no one shall soil our sheets but ourselves.

Now, although we may not have the same antipathy to bad grammar that W. *seems* to have, nor that aversion to being "scolded in bad grammar by a *professor*" that W.'s boy had; still we dont like it in *toe-toe;* nor yet as *thick* as we find it in W.'s spicy article under that witty heading, and we hope—and will kindly suggest, that in future he will "draw it mild."

